# Joint Tuberculosis

**Published:** 1911-09

**Authors:** 


**Joint Tuberculosis.** By **Leonard W. Ely,** M. D., Consulting Orthopedist to the County Hospital; Attending Orthopedist to the Children's Hospital, Denver. 8 mo, pp. 243. Illustrated. New York: William Wood & Co. 1911. (Cloth, $2.50.)

The book is timely and concisely written. The author, an orthopedic surgeon, has had much to do with joint tuberculosis, and is to be complimented on his success in combining the pathological and practical experience on this important subject. He has not only given his own ideas as to radical methods, but has thoroughly reviewed the literature on this subject, stating frankly the opinions of other authors. The etiology, pathology, symptomatology, diagnosis, prognosis and treatment are fully taken up in the various chapters under these headings. Many original ideas have been advanced. It is not the intention of the book to take up detailed treatment of individual joints, nor to include all of the orthopedic measures necessary to control tubercular joints.

In short, the book is a very excellent treatise on the subject, with valuable references and comparisons throughout.

